# Optimizing 1D-CNN-Based Emotion Recognition Process through Channel and Feature Selection from EEG Signals

**DOI:** 10.3390/diagnostics13162624

**Published:** 2023-08-08

**Authors:** Haya Aldawsari, Saad Al-Ahmadi, Farah Muhammad

**Affiliations:** 1 Department of Computer Science, College of Arts and Science, Prince Sattam bin Abdulaziz University, Al-Kharj 16278, Saudi Arabia; hs.aldawsari@psau.edu.sa; 2 Center of Excellence in Information Assurance (CoEIA), King Saud University, Riyadh 11543, Saudi Arabia; salahmadi@ksu.edu.sa; 3 College of Computer & Information Sciences, King Saud University, Riyadh 11543, Saudi Arabia

**Keywords:** emotion recognition, EEG, 1D-CNN, human-computer interactions

## Abstract

EEG-based emotion recognition has numerous real-world applications in fields such as affective computing, human-computer interaction, and mental health monitoring. This offers the potential for developing IOT-based, emotion-aware systems and personalized interventions using real-time EEG data. This study focused on unique EEG channel selection and feature selection methods to remove unnecessary data from high-quality features. This helped improve the overall efficiency of a deep learning model in terms of memory, time, and accuracy. Moreover, this work utilized a lightweight deep learning method, specifically one-dimensional convolutional neural networks (1D-CNN), to analyze EEG signals and classify emotional states. By capturing intricate patterns and relationships within the data, the 1D-CNN model accurately distinguished between emotional states (HV/LV and HA/LA). Moreover, an efficient method for data augmentation was used to increase the sample size and observe the performance deep learning model using additional data. The study conducted EEG-based emotion recognition tests on SEED, DEAP, and MAHNOB-HCI datasets. Consequently, this approach achieved mean accuracies of 97.6, 95.3, and 89.0 on MAHNOB-HCI, SEED, and DEAP datasets, respectively. The results have demonstrated significant potential for the implementation of a cost-effective IoT device to collect EEG signals, thereby enhancing the feasibility and applicability of the data.

## 1. Introduction

Emotion is defined as the reaction to or consciousness of external stimuli, playing an important role in daily life by affecting people’s routines. Basic emotions like happiness, anger, and sadness are constantly reflected by people voluntarily or involuntarily, significantly impacting their position in society. Negative emotions can lead to social exclusion, causing physiological and psychological effects [1]. Positive emotions relate to better living standards and a longer life [2]. Many studies have analyzed emotions, with recent ones focusing on understanding emotional behaviors [3,4]. However, the abstract and individualized nature of emotions makes this research challenging and limits progress [5]. Moreover, the multitude of data collection methods (facial, vocal, neural, bodily signals, etc.) and analysis techniques creates complex, time-consuming data processing [4]. Therefore, computer-aided AI-based analysis methods are needed. In summary, emotion analysis is critical but faces difficulties due to the inherent complexity of emotions. Advanced computational techniques are thus necessary to enable effective emotion research.

EEG-based emotion recognition has wide applications but can identify hidden internal states beyond external expressions. EEG directly measures brain signals using scalp electrodes, capturing real-time data on unspoken emotions. This enables intuitive responses to users’ psychological states in diverse systems. Therefore, this study has utilized EEG data to recognize emotions. The process of emotion recognition typically involves three main steps: preprocessing, feature extraction, and classification. Pre-processing procedures are essential for data preparation prior to further analysis. Feature extraction can be achieved using traditional machine learning or deep learning methods. Traditional methods often involve the use of handcrafted features [6]. However, owing to the high non-stationarity of EEG signals, extracting such features is challenging and requires expert knowledge. Recent studies have explored multivariate statistical analysis techniques in the frequency, time-frequency, and nonlinear domains that effectively represent EEG characteristics [7,8,9]. For instance, Zheng et al. [7] evaluated different feature extraction methods and achieved the best average accuracy using a discriminative graph-regularized Extreme Learning Machine with differential entropy features. Zhu et al. [8] constructed a graph structure based on differential entropy characteristics, learned channel relationships through dynamic simplifying graph convolutional networks, and recalibrated channel features using the style recalibration module. Through the fusion and classification of sub-band features, the method achieves improved classification accuracy compared to existing methods. Wang et al. [9] proposed an end-to-end convolutional neural network (CNN) model called SACNN to improve cross-subject emotion recognition accuracy. By restructuring the data dimensions and selecting the top 10 channels, the SACNN model achieved significant accuracy in detecting human emotions. Algarni et al. [10] proposed a stacked bi-directional Long Short-Term Memory (Bi-LSTM) Model for EEG-based emotion recognition that includes Binary Gray Wolf Optimizer for feature selection. By achieving high accuracy in classifying arousal, valence, and liking from the DEAP dataset, the proposed method demonstrated effective emotion recognition through EEG signals. Li et al. [11] proposed a C-RNN model combining CNN and RNN to identify emotions using multichannel EEG signals. While RNN-based methods have advantages for processing time series data and have achieved great results, they still have limitations when dealing with multichannel EEG data. Gao et al. [12] proposed a gradient particle swarm optimization (GPSO) model to automatically optimize CNN models. They implemented the GPSO algorithm to optimize the hyperparameters and architecture of CNNs. The experimental results demonstrated that CNN models optimized by their proposed GPSO approach achieved significantly higher classification accuracy compared to other CNNs. Hancer and Subasi [13] proposed a unique EEG emotion recognition framework including multi-scale PCA and wavelet filtering for preprocessing. For feature extraction, they used dual tree complex wavelet transform. For feature selection, they employed statistical criteria to reduce dimensions. Consequently, researchers have been utilizing various neural networks for EEG data analysis [14,15,16] with the aim of improving accuracy.

EEG-based emotion recognition tasks using deep learning models face a significant challenge due to the limited availability of EEG training datasets compared to visual and audio datasets. Currently, only a few public datasets, including MAHNOB-HCI [17], DEAP [18], and SEED [19], are accessible for EEG-based emotion recognition, and their scale is small to train a deep learning model efficiently [20]. To overcome this limitation, data augmentation techniques have been employed to generate additional training data. These techniques involve applying geometric modifications or adding Gaussian noise to the original EEG data [16]. However, a recent approach by Kalaganis et al. [21] executes a graph variant of the classical Empirical Mode Decomposition (EMD) to produce realistic EEG-like data. The generated data, combined with the original data, is then utilized to train a 1D-CNN model for classification and an Arousal-Valence model to identify emotions from complex and nonstationary EEG data. Experimental results have demonstrated that data augmentation significantly enhances the accuracy of classifiers in EEG-based emotion recognition.

The cerebral cortex has four lobes: frontal, parietal, temporal, and occipital. EEG devices vary in electrode number, categorized as low (1–32 electrodes), medium (33–128), or high (>128) resolution [22]. The international 10–20 system [23] standardizes electrode placement by mapping locations to scalp regions. Selecting optimal EEG channels for emotion recognition from key brain areas can reduce the electrodes needed, improving wearable headset comfort. Determining these optimal channels has been a focus for enabling real-world EEG applications. The frontal, parietal, temporal, and occipital lobes each contribute unique signals relevant for emotion recognition. By exploring the most informative regions and channels, this work aims to provide insights on channel selection to reduce computational demands while preserving emotion-relevant information from EEG signals. The goal is to improve the feasibility of EEG-based emotion recognition.

Previous research has recognized human emotions using a limited number of EEG channels. While this can increase computational speed, it decreases the accuracy of emotion recognition. In this paper, we introduced a differential entropy-based approach for channel selection that not only enhances the model’s performance but also reduces the reliance on using all EEG electrodes for signal extraction by selecting the most relevant channels. Moreover, challenges remain in computational efficiency, robust feature extraction, and optimal model architectures. Continued research is needed to develop end-to-end systems that leverage different deep networks’ complementary strengths with a fewer number of channel EEG analyses. Therefore, the paper utilizes a Decomposed Mutual Information Maximization (DMIM) method for sequential feature selection, which eliminates unwanted data from the selected channels to further improve the model’s performance. Furthermore, it proposes a lightweight 1D-CNN architecture based on deep learning for the classification of high-quality data obtained from selected EEG channels. Lastly, the paper incorporates a graph-EMD to generate realistic EEG-like data, enhancing the training dataset.

The remaining sections of the paper are organized as follows: Section 2 provides a detailed description of the research methodology, specifically the presented deep learning model. In Section 3, the results of the analysis are presented. Finally, Section 4 and 5 offer a discussion of the work and conclude the paper, respectively.

## 2. Materials and Methods

**Figure 1:**
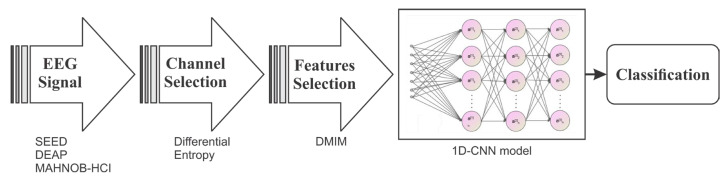
Emotion recognition process using proposed framework.

In this section, we provide a comprehensive overview of the MAHNOB-HCI, SEED, and DEAP datasets, along with the pre-processing procedures employed. Following that, we delve into the detailed introduction of our model. Lastly, we elucidate the training parameters utilized in our model for a comprehensive understanding of the training process. Figure 1 illustrates the process of proposed framework.

### 2.1. Dataset and Data Processing

The MAHNOB-HCI database [17] contains EEG and physiological signals recorded from 30 participants (data from 3 subjects was lost) watching 20 emotional video clips. The 17 women and 13 men healthy adult participants were 19–40 years old. These participants watched 20 emotional video clips chosen to elicit responses of disgust, amusement, fear, sadness, and joy. The clips were selected from movies and online videos. The clips ranged from 34 to 117 s, with a 30 s baseline before/after each clip. EEG data were recorded using caps with 32 electrode sensors and sampling rate of 512 Hz, which was then down-sampled to 256 Hz. After each video, participants self-reported their arousal and valence using the Self-Assessment Manikin (SAM) scale. Arousal measures the degree of excitement, rated from 1 (boring) to 9 (exciting), and valence indicates the polarity of emotion, rated from 1 (unpleasant) to 9 (pleasant) [24]. Therefore, the dataset includes subjective arousal and valence ratings from 1 to 9 for each video based on the participants’ emotional responses. These self-reported scores provide ground truth labels to relate the recorded EEG and physiological signals to emotional states defined along the dimensions of arousal and valence. In total, the database includes EEG and peripheral signals for emotion analysis from 27 subjects responding to 20 videos.

The DEAP dataset [18] contains EEG recordings from 32 participants (16 male, 16 female) as they watched 40 one-minute musical video clips. Each trial included a 3 s pre-trial baseline followed by 60 s of video stimulation. EEG data were recorded using caps with 32 electrode sensors and sampling rate of 512 Hz. The data were preprocessed by the DEAP team, including down-sampling the EEG signal to 128 Hz. After each video, participants self-reported their arousal valence, like/dislike, dominance, and familiarity levels using the SAM scale. Each subject underwent 40 trials, with various signals recorded as 40-channel data. The first 32 channels represented EEG signals, while the remaining 8 channels captured autonomous physiological signals. After watching each video, participants rated the levels of arousal, valence, like/dislike, dominance, and familiarity using SAM.

The SEED dataset [19] consists of EEG signal data obtained during an emotional experiment. It was collected from 15 subjects, comprising 7 males and 8 females. Sixty-two-channel EEG data were collected as participants viewed 15 four-minute Chinese film excerpts designed to elicit positive, neutral, and negative emotions (5 clips per emotion). During the experiments, subjects received a 5-s prompt before each clip, then completed a 45-s self-assessment of their emotions followed by 15 s of rest. The dataset focused on three specific emotions: positive, neutral, and negative, resulting in a total of 45 trials. Each trial involved recording the scalp EEG signals using a 62-channel standard 10–20 system at a sampling rate of 200 Hz, which was then down-sampled to 128 Hz. To eliminate physiological noises, a 3rd-order bandpass Butterworth filter with cutoff frequencies of 0 Hz to 75 Hz was applied. A comparison between the various characteristics of all the datasets under consideration has been depicted in Table 1.

For this work, the valence and arousal scales were used for classification. The SAM rating scale was divided into two parts, high (level > 5) and low (level ≤ 5), to establish low valence (LV), high valence (HV), low arousal (LA), and high arousal (HA) labels for MAHNOB-HCI and DEAP datasets. However, for SEED dataset, three classes (positive, neutral, and negative) were considered for classification.

### 2.2. Data Augmentation

In recent years, there has been growing scientific interest in applying data augmentation for EEG classification. George et al. [25] performed data augmentation for EEG signals using six different synthesis approaches. The results showed that the synthesized EEG data exhibited similar characteristics to real data. Using the augmented data for training increased classification accuracy by up to 3% and 12% on two public EEG datasets. Luo et al. [26] used conditional Wasserstein GAN (cWGAN) and selective VAE (sVAE) for data augmentation to improve emotion recognition from EEG signals. They transformed the training data into power spectral density and differential entropy representations before feeding them into the cWGAN and sVAE models. Their proposed augmentation technique helped in improving classification performance for EEG-based emotion recognition. Zhang et al. [27] proposed a new EEG data augmentation method using empirical mode decomposition (EMD). In their approach, EEG signals are decomposed into intrinsic mode functions (IMFs) using EMD. The IMFs are then recombined in different ways to artificially generate new training data. This augmented data are transformed into wavelet tensors and used to train a convolutional neural network (CNN) classifier. Experiments showed that their EMD-based data augmentation technique improved EEG classification accuracy compared to training without data augmentation. Abdelfattah et al. [28] proposed recurrent generative adversarial networks (RGANs) to generate synthetic EEG. The recurrent generator architecture allowed the RGANs to successfully capture the key temporal dynamics of EEG signals, giving them an advantage over standard GANs for EEG data generation. To address limited dataset sizes for EEG-based emotion recognition, this study explored using graph empirical mode decomposition (EMD) for augmenting EEG signals to improve classification.

The graph variant of the classical EMD method allows the generation of artificial EEG epochs using an arbitrary number of input epochs. To generate these artificial epochs, each individual EEG epoch is initially decomposed into a set of graph Intrinsic Mode Functions (IMFs) based on a graph structure G. By combining the graph IMFs from different epochs, an artificial EEG epoch can be created. This approach leverages the mono-component nature of the graph IMFs, resulting in artificial epochs that share similar characteristics with the original signals that contributed to their IMFs.

To enhance the capabilities of a classifier, the class information associated with each EEG epoch is considered during the generation of artificial epochs. Consequently, the corresponding IMFs of each epoch are also assigned the same class label, ensuring that each artificial EEG epoch is generated exclusively from graph IMFs belonging to a single class. This approach enables the creation of artificial EEG epochs that align with specific class characteristics, further enhancing the discriminative capabilities of the classifier.

The proposed data augmentation method as discussed by Kalaganis et al. [21] involves the following steps to generate artificial EEG epochs:

1. Random selection of class-specific EEG epochs: For each class, a random set of EEG epochs is chosen to contribute their Intrinsic Mode Functions (IMFs). The number of contributing EEG epochs is determined by the maximum number of IMFs present in a signal segment.
2. IMF selection for artificial EEG epoch generation: To create an artificial EEG epoch, the IMFs are selected in a sequential manner. The first IMF is taken from the first contributing EEG epoch, the second IMF from the second contributing EEG epoch, and so on. If a contributing EEG epoch has fewer IMFs than required, the additional IMFs are considered as zero graph signals. The required maximum number of IMFs is set to five.

By following this procedure, a substantial number of artificial EEG epochs can be generated, with the potential to reach the total number of EEG epochs raised to the power of the number of graph IMFs. This augmentation process effectively expanded the dataset, providing a greater variety of data samples for analysis and training purposes.

### 2.3. Proposed Architecture

#### 2.3.1. Channel Selection

According to neuroscience, the brain waves are generated in specific regions of the brain. In particular, the pre-frontal, parietal, and temporal areas are associated with the detection of positive valence emotions [29]. Several recent studies have explored EEG channel selection for emotion recognition. Affes et al. [30] proposed a cascading deep learning model called CAtt-MLP for channel selection, consisting of convolutional blocks, an attention neural network, and a multi-layer perceptron (MLP). Ramadhani et al. [31] applied integrated selection to remove irrelevant channels, improving brain–computer interface performance. Dura and Wosiak [32] used a reversed correlation algorithm (RCA) to automatically select optimal EEG electrodes. RCA identified emotion-relevant band-channel combinations through inter-subject analysis. Daoud and Bayoumi [33] used transfer learning and semi-supervised approaches to identify relevant channels. According to previous research [34,35], the most commonly used electrodes for emotion recognition are O1, O2, FP1, FP2, F3, F4, C3, C4, AF3, AF4, FC5, FC6, T7, T8, F7, F8, P7, and P8. Moreover, the frontal lobe electrodes like F3, F4, and AF3/4 have been used in more than 70% of the studies, which shows that many researchers believe the frontal cortex plays an important role in emotion processing. Across these works, channel selection consistently improved accuracy compared to using all channels. By removing less informative channels, channel selection reduces complexity and noise while focusing on emotion-relevant EEG features.

In this work, we have employed the concept of differential entropy to select the channels. By exploiting this measure, we can identify and incorporate the relevant channels in this work. Formally, let (*U*, *C*∪*D*) be an informed decision, where *U* is the set of all EEG data samples under consideration, categorized into two classes *C* and *D*. For a particular EEG sample *P* belonging to class *C* (*P* ⊂ *C*), the differential entropy *E* of *P* with respect to class *C* is defined as:

(1) $$E\left(P|U⊕C\right)=-\frac{1}{U}\sum_{z\in U}\log_{2}\frac{\left|{\left[x\right]}_{C}\cap {\left[x\right]}_{P}\right|}{\left|{\left[x\right]}_{P}\right|},$$

**Figure 2:**
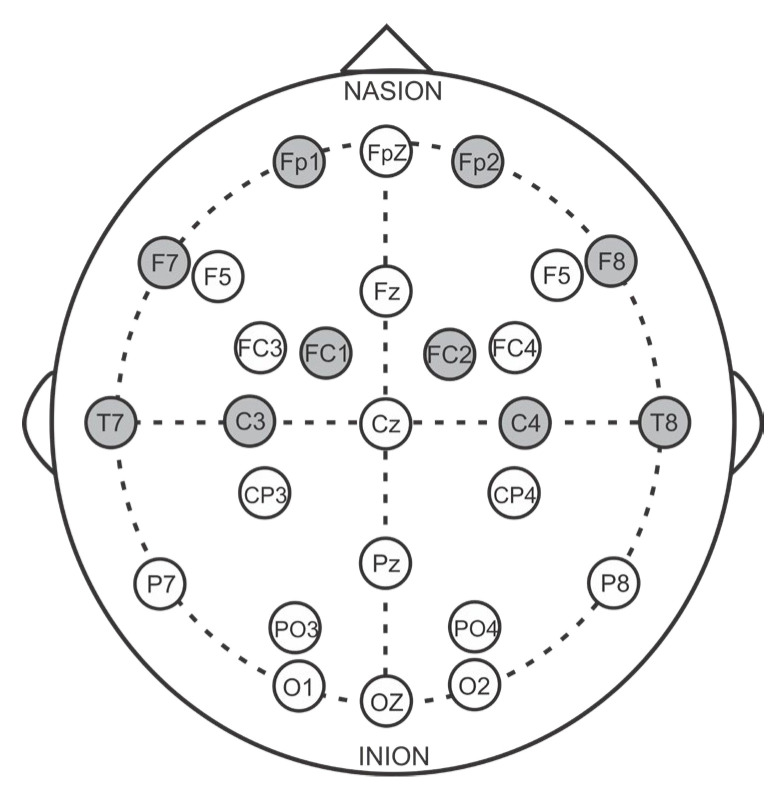
Selected most significant channels for class discrimination are the ones with filled circles.

We conducted a series of experiments using different threshold values between 1.0 and 2.5 to evaluate the performance of emotion recognition when using selected channels, with the help of differential entropy, as input to the proposed 1D-CNN model. Although the model performance was sufficiently accurate with several channel numbers, we tried to optimize channel selection across all datasets under consideration to find the best performing combination. Therefore, a threshold of 1.5 was chosen to select the most significant EEG channels that directly impacted class discrimination. This threshold value not only helped achieve the same number of channels across the SEED, DEAP, and MAHNOB-HCI datasets but also improved emotion recognition performance using a limited number of channels. Specifically, 10 common channels were selected from the pool of most significant channels for all three datasets, as shown in Figure 2. Using this data-driven approach to select the optimal channels enabled improved emotion recognition with a small subset of EEG channels that were most informative for discrimination. The threshold of 1.5 struck the right balance between channel reduction and preserving class-discriminative information.

#### 2.3.2. Feature Selection

Feature selection is a well-established preprocessing technique that plays a crucial role in reducing dimensionality and enhancing the performance of regression and classification tasks. Among the various approaches, the sequential forward feature selection methods based on Mutual Information (MI) have gained popularity in practical applications due to their computational efficiency and classifier independence. In our study, we have specifically focused on the Decomposed Mutual Information Maximization (DMIM) method [36]. This method retains the desirable theoretical properties of the most effective existing methods while addressing the issue of complementarity penalization. By separately maximizing the inter-feature and class-relevant redundancies, DMIM overcomes this limitation and offers improved performance.

For this purpose, we first divided the entire signal of each selected channel into multiple samples. We achieved this by applying a 2-s window without overlapping, allowing for better evaluation. Additionally, we labeled each sample based on its position in the original signal before applying differential entropy. This labeling enables us to obtain the original signal of each sample after ranking them. We then applied differential entropy to each 2-s sample using the following Formula (2) to use it for DMIM-based feature ranking method:

(2) $$h\left(x\right)=\frac{1}{2}\log\left(P\right)+\frac{1}{2}\log\left(\frac{2\pi e}{N}\right),$$

where *P* represents the average energy value of the EEG signal by replacing the variance, and the *N* represents the length of fixed time window, which is 2 s in this case.

Afterwards, we utilized the DMIM based features ranking method described by [36], which maximizes inter-feature and class-relevance redundancy separately, enabling joint optimization of feature diversity and relevance for selection. The mutual information between two variables can be calculated as follows:

(3) $$MI\left(A,B\right)=\underset{a=𝒶}{\sum }\underset{b=𝒷}{\sum }P\left(A=a,B=b\right)ln\frac{P\left(A=a,B=b\right)}{P\left(A=a\right)P\left(B=b\right)},$$

Therefore, suppose $X_{i}\in F$ be the feature being used for prioritization and its relevance with the class *C*; the objective function *DMIM* using *MI*(.) can be defined as follows:

(4) $$DMIM\left(X_{i}\right)=MI\left(X_{i},\;C\right)-\underset{X_{s}\in S}{\max}MI\left(X_{i},\;X_{s}\right)+\underset{X_{s}\in S}{\max}MI(X_{i},\;X_{s}|C),$$

Each term of the objective function *DMIM* states a unique type of contribution between the class (*C*) the set of selected features (***S***) and the features ($X_{i}$), whereas $X_{s}\in S$ are the selected features. The first term *MI*(.) measures the relevance of feature $X_{i}$ to the class. The second term *maxMI*(.) measures redundancy between $X_{i}$ and $X_{s}$. The third term *maxMI*(.) measures redundancy between $X_{i}$ and the class-specific information in selected features. Overall, the three terms quantify relevance, inter-feature redundancy, and class-conditional redundancy for feature selection.

**Figure 3:**
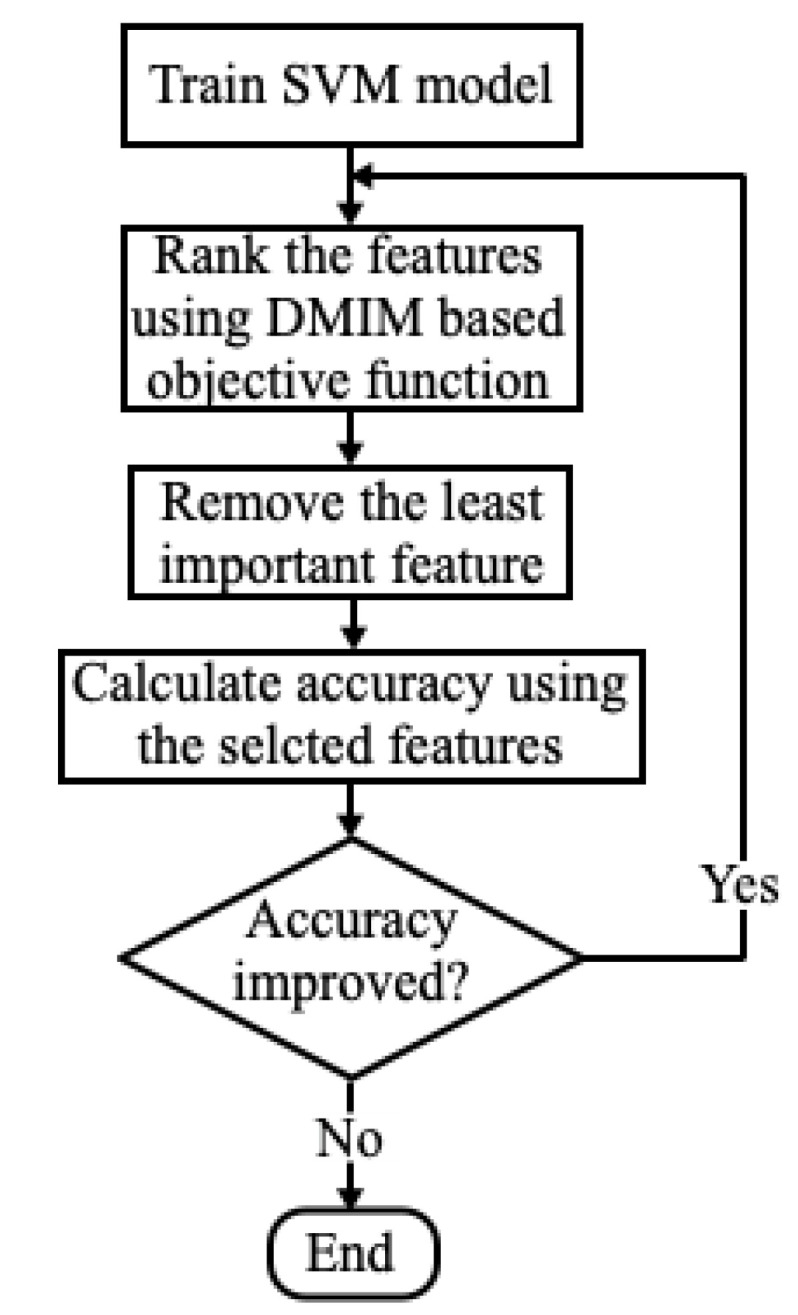
Process of selection of features using DMIM based objective function.

Our complete evaluation process for feature selection is provided in Figure 3 in terms of flow chart. While the accuracy of recognition is increasing, the process of feature reduction continues until the accuracy does not improve further. In this way, the highly ranked features are selected and contain class-specific information while removing redundant features.

**Figure 4:**
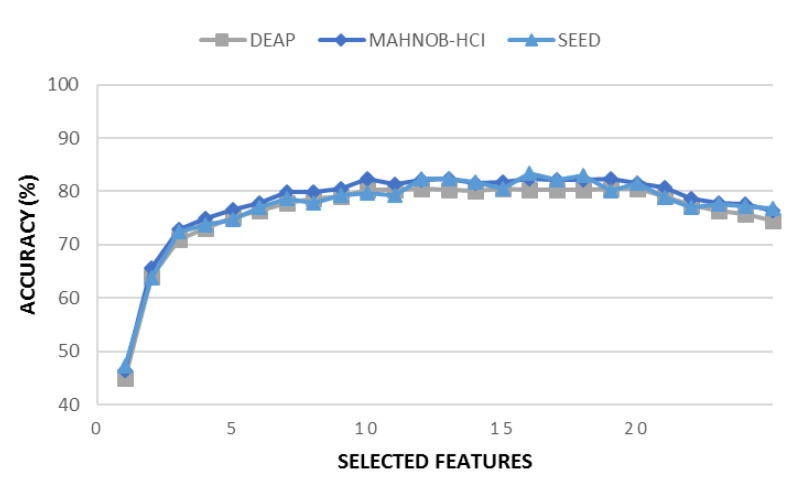
A plot between accuracy and selected features of a particular selected channel using DEAP, MAHNOB-HCI and SEED datasets.

Once we obtained the feature ranking from *DMIM*, we selected only the top twenty features with the highest contribution to class discrimination with respect to classification accuracy and disregarded the remaining ones. On average, the first twenty features had a greater influence on the accuracy of emotion recognition compared to the rest of the features (see Figure 4). Therefore, we chose these twenty features as the high-quality features. Subsequently, we obtained the original EEG samples corresponding to these features for further use in the next stage of the method, which is the 1D-CNN model. The typical size of feature vectors obtained from each level are listed in Table 2.

#### 2.3.3. 1D-CNN Model

The extracted high-quality features from the original EEG data are passed through the 1D-CNN model. The model consists of multiple pairs of convolutional, batch normalization and pooling layers, as well as an output layer. Prior to being input into the 1D-CNN model, the features are combined into a vector format, which is subsequently convolved with a set of one-dimensional filters within the convolutional layers. Following the pooling layer, the data undergo further down-sampling, resulting in vectors with reduced dimensions. The network weights and filters within the convolutional layers are trained using the back-propagation algorithm.

The CNN structure employed in this study is relatively straightforward. The input vector is a one-dimensional feature represented by *X*, with a shape of *N* × 1. At the convolutional layer, the input feature vector is convolved with filters *W_f_*. The convolution operation results in the formation of an output map, and the feature map at the corresponding layer is obtained using the equation provided below:

(5) $$f\left(\alpha \right)=f\left(W_{f}X+b_{f}\right),$$

where, weight matrix $W_{f}\in R^{i\times 1}$, and the bias value *b* are utilized in the calculation, with *f* denoting the filter index ranging from 1 to *n*, where *n* represents the total number of filters in the convolutional layer. In this work, the activation function employed is the rectified linear unit (ReLU), denoted by the function *f*. ReLU is more efficient in preventing gradient disappearance compared to traditional neural network activation functions like *sigmoid* and *tanh*. The ReLU function is defined as follows:

(6) $$f\left(\alpha \right)=\mathrm{ReLU}\left(\alpha \right)=\ln\left(1+e^{\alpha }\right),$$

where value of *α* has been defined in previous equation.

Batch normalization is a well-known technique in modern neural networks that serves two purposes: normalizing the output from the previous layer in a CNN model and regularizing the data to prevent overfitting. It plays a crucial role in ensuring stable training and enhancing the generalization capability of the network. At the max-pooling layer, the feature map undergoes down-sampling through the application of an average-pooling function. This function is employed because it has been observed that extracting the average value from the selected values within a given feature map is an effective approach. This significantly reduces the number of trainable parameters, leading to an accelerated training process. Also, the dropout layer is employed to prevent overfitting.

Following the last pooling layer, a fully connected layer is employed, where the output data from the pooling layer is flattened. Subsequently, a series of fully connected layers called DNN (Deep Neural Network) is utilized. In each layer of the DNN, the activation function employed is also ReLU.

**Figure 5:**
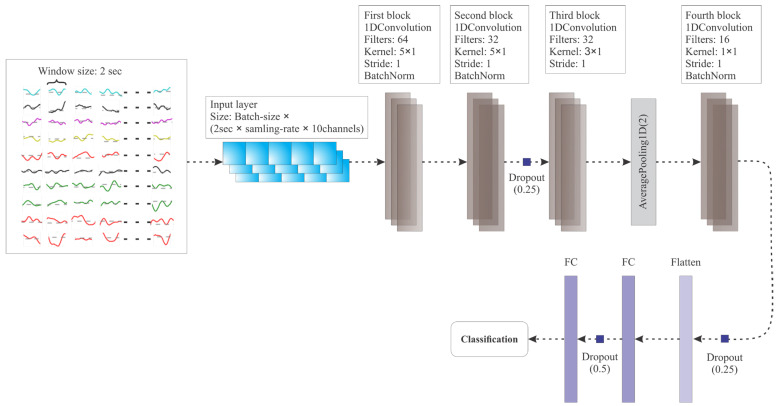
Architecture of Proposed 1D-CNN model. Note. The different colors of input EEG samples at each row shows a signal from different channel.

For the output layer, two classification tasks are considered: binary classification and three-class classification. To address the binary classification task (for DEAP and MAHNOB-HCI dataset), the sigmoid function is utilized as an activation function, while for the three-class classification task (for SEED dataset), the softmax function is employed. Additionally, for the binary-classification task, the Adadelta optimizer is used, and the loss is calculated using the binary cross-entropy formula, as indicated by the equation below:

(7) $$loss_{CE}=-\sum_{n=1}^{N}{\hat{y}}_{i}\log\left(y_{i}\right)+\left(1-{\hat{y}}_{i}\right)\log\left(1-{\hat{y}}_{i}\right),$$

where *N* represents the number of samples, $y_{i}$ denotes the value in the encoded form, and ${\hat{y}}_{i}$ represents the output obtained from the output layer using sigmoid activation. In the case of three-class classification, Adam optimizer is employed, and the loss is calculated using categorical cross-entropy, which can be expressed as

(8) $$loss_{CCE}=-\sum_{n=1}^{N}{\hat{y}}_{i1}\log\left(y_{i1}\right)+{\hat{y}}_{i2}\log\left({\hat{y}}_{i2}\right)+{\hat{y}}_{i3}\log\left({\hat{y}}_{i3}\right),$$

where *N* represents the number of samples, and $y_{i1}$, $y_{i2}$, and $y_{i3}$ correspond to the values of the label, which are encoded. The values ${\hat{y}}_{i1}$, ${\hat{y}}_{i2}$, and ${\hat{y}}_{i3}$ represent the three outputs obtained from the output layer, where softmax activation is applied. The model’s parameters are updated using the back-propagation algorithm. The error between the desired output and the actual output is computed, and the gradient descent method is utilized to update the parameters, aiming to minimize the error. The weight and bias update functions are illustrated as follows:

(9) $$W_{f}=W_{f}-\eta \frac{\partial E}{\partial W_{f}},\;\;b_{f}=b_{f}-\eta \frac{\partial E}{\partial b_{f}},$$

where *W_f_* refers to the weight matrix, *b_f_* represents the bias, *η* denotes the learning rate, and *E* represents the error, which is the loss calculated in Equations (7) and (8). An illustration of the proposed 1D-CNN model has been provided in Figure 5.

#### 2.3.4. Hyperparameters

In our study, we utilize a 1D-CNN to enhance the capture of EEG signal details linked to emotions. A grid search approach was considered to achieve the most suitable set of hyper-parameters of this 1D-CNN model. Table 3 presents the key hyperparameters, their corresponding range of values, and selected values for the proposed model in this study.

The model is implemented using Python 3.9. As depicted in Figure 5 and Table 2, the size of the input 1D vector comprises of 10 (EEG channels) × 2 (window size: 2 s) × 128 (sampling rate: 128 Hz (for DEAP and SEED datasets)/256 Hz (for MAHNOB-HCI dataset)). The batch size for the model is set to 64, resulting in input data shapes of (163,840) for the DEAP and SEED datasets and (327,680) for the MAHNOB-HCI dataset.

In the proposed model, the term “1DConvolution” represents the one-dimensional convolutional layer, “AveragePooling1D” denotes one-dimensional average pooling, “BatchNorm” refers to one-dimensional batch normalization, and “FC” represents the fully connected layer. Each convolutional layer in the model is followed by an activation layer, where ReLU is used as the activation function.

The proposed model consists of four convolutional layers, three batch normalizations, two dropout layers with probabilities of 0.25, one pooling layer, one flatten layer, and two fully connected layers. These layers are combined to form four convolution blocks. The first block contains a convolutional layer with a kernel size of 5 × 1, aimed at extracting emotion-relevant features. *ReLU* is used as the activation function for each convolutional layer. Each convolution block, except for one, includes a normalization layer. The third convolution block is connected to an average pooling layer at the end. The last convolution block is followed by a fully connected layer, which is then connected to the final output fully connected layer after a dropout layer with a probability of 0.5. The final output of the model provides the classification results for emotion recognition. The structure of the proposed model is illustrated in Figure 5.

## 3. Results

In this section, we analyze the implementation of the proposed feature selection method by utilizing the classification results obtained through our approach. We have conducted various evaluation parameters to validate the results and compared them with those obtained from the state-of-the-art models to verify the effectiveness of the proposed method. To mitigate the issue of overfitting, the dataset was typically partitioned into random subsets of equal size. Accordingly, we employed the leave-one-subject-out strategy to divide our dataset for training and testing sets.

We classified three (positive, neutral, negative) emotional states for the SEED dataset and two (HV, LV) and (HA, LA) emotional states for the DEAP and MAHNOB-HCI dataset using the designed 1D-CNN model. The accuracy of the selected features for each subject in the datasets was used to validate the results. To calculate the accuracy, precision, specificity, recall, *F*1-score, and *p*-score, we employed the confusion matrix and utilized the keywords True positive, False positive, True negative, and False negative. The performance metrics for the DEAP, MAHNOB-HCI, and SEED datasets are presented in Table 4, Table 5 and Table 6, respectively.

Moreover, a *t*-test was conducted to evaluate the *p*-score (probability score), aiming to assess the usefulness of the selected function. It determined whether there existed a significant difference between the selected feature vector and the original or actual feature vector. The *p*-value was derived by calculating the mean difference between the selected objects and the original object vector. A higher *p*-value indicated a higher quality factor for the selected item.

**Figure 6:**
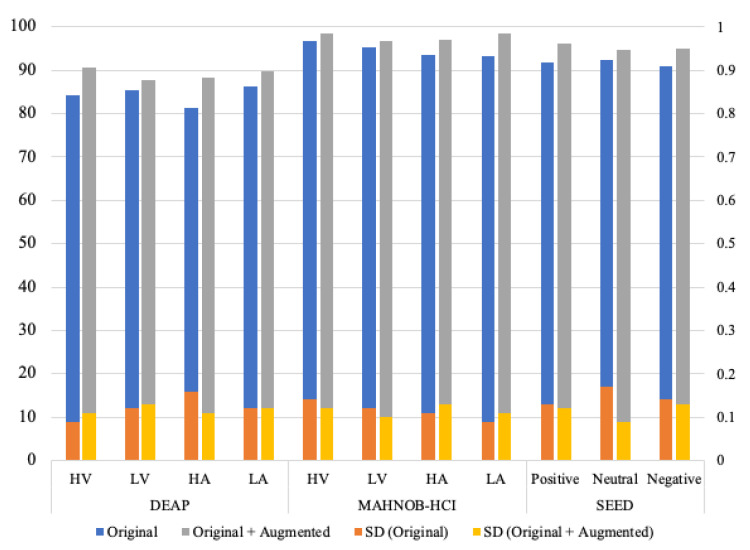
Performance (average accuracy/standard deviation (SD)) of the proposed model with/without augmented data using graph-EMD. The *Y*-axis on the left side shows the average accuracy associated with the Original and Original+Augmented data, while the *Y*-axis on the right side shows the values of the SD associated with the respective average accuracies.

Additionally, we performed data augmentation technique to increase the number of samples. Usually, deep learning models require a large amount of datasets to produce better results. Therefore, we added the same number of generated samples of EEG data to the original datasets of DEAP, MAHNOB-HCI, and SEED, respectively. The results of adding fake data using graph-EMD have been presented in Figure 6 with class-specific accuracies. The average accuracies are valued on the left side of the *Y*-axis, while the values of the standard deviation (SD) associated with the respective average accuracies are represented on the right side of the *Y*-axis. As shown in the figure, the LA class of the MAHNOB-HCI dataset attained the highest average accuracy of 98.43% with 0.11 SD using augmented data. The least SD of 0.09 is attributed to the neutral class of the SEED dataset with an average accuracy of 94.65% with augmented data.

**Figure 7:**
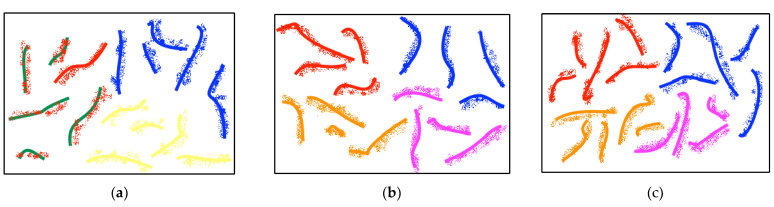
The t-SNE visualization of the real and generated EEG data distributions for one subject from the SEED, DEAP, and MAHNOB-HCI datasets is shown in (**a**–**c**), respectively. The lines represent the real EEG data, while the scattered points are the artificially generated augmented data. In (**a**), the data points colored red, blue, and yellow represent the negative, positive, and neutral classes, respectively. In (**b**,**c**), the data points colored red, orange, blue, and magenta represent the high valence, low valence, high arousal, and low arousal classes, respectively.

To illustrate the benefits of data augmentation using graph-EMD, we visualized the real and synthesized samples through t-SNE two-dimensional projections, as shown in Figure 7. From the results, we can observe that (1) the generated data matches the actual data distributions in each dataset and (2) the augmented data maintains class-specific properties as expected, without low-quality samples that could mislead the classifier. Overall, the t-SNE visualizations demonstrate that the synthesized data has similar distributions to the real data for each emotion class, validating the quality of the graph-EMD augmentation approach.

### Performance Analysis

The computational time taken by the proposed model with and without the channels and the attributes selected through the proposed method has been calculated. The following equation has been used to calculate the time [37]:

(10) $$CT=\frac{1}{F_{T}}\sum_{c_{i}=1}^{C}\left(\left[C_{P}\times \ln\left(f_{v}\right)\right]+\left[\left(1-C_{P}\right)\times \ln\left(1-f_{v}\right)\right]\right),$$

where the selected feature value is denoted as *f_v_*, the training features are depicted as *F_T_*, the features of a particular emotional category are specified as *C_P_*, the number of emotional categories is symbolized as *C*, and the categories index is symbolized as *c_i_*.

The processing time is reduced as a result of selecting a smaller number of features. By employing the differential entropy for channel selection and the feature reduction method using DMIM, emotions can be recognized within a shorter processing time while maintaining the overall classification performance. Table 7 presents the execution time of the proposed method, comparing the results with and without the channel/feature selection method.

The channel selection greatly enhances the overall rating performance, as depicted in Table 8, where the selected channel outperforms all other channels. This improvement can be attributed to the fact that not all channels provide high-quality stimuli for emotions in EEG signals. This study highlights the importance of channel selection and its impact on understanding the emotional behavior captured by the selected channel. Also, a comparison of average accuracies on augmentation has also been provided in Table 8. It shows the efficiency of the proposed method with and without augmentation. Moreover, we used the Kappa coefficient to evaluate emotion recognition accuracy. Kappa measures the agreement between predicted and true labels, normalized for chance and classifier count. Unlike basic accuracy, Kappa provides a standardized performance estimate adjusted for variations in classes and classifiers.

## 4. Discussion

Given the important role of emotion in human–machine interactions, this study aims to recognize emotions using EEG signals captured with a limited number of electrodes. While people may more easily identify specific emotions they feel, the dimensional perspective offers a more fundamental understanding of the emotion–behavior relationship [38]. Neuroscience studies also support this dimensional view. Evidence of neural activities shows that valence and arousal influence cognition and behavior through distinct brain mechanisms [39]. Consistent with this theoretical background, this study systematically manipulated and examined valence and arousal effects rather than focusing on discrete emotions. By taking a dimensional approach to emotion recognition from limited-channel EEG data, this work provides insight into the fundamental mechanisms linking EEG signals to emotional states.

We have introduced a deep learning-based 1D-CNN model that effectively utilizes differential entropy for channel selection and the DMIM method for feature selection. This model efficiently processes raw EEG signals to recognize emotions, enhancing the visibility and efficiency of class-specific features. The proposed architecture simultaneously extracts valuable EEG patterns, considering the recent literature emphasizing the significance of channel selection in emotion recognition. Consequently, we specifically employ differential entropy to identify the most relevant channels for emotion recognition. Furthermore, we employ the DMIM method to select high-quality features, dividing the EEG signal into samples of 2 s on a horizontal scale. This helped in assessing the signal in a better way on a temporal scale and reducing the feature vector for the deep learning model.

In Table 4, Table 5 and Table 6, we provide the evaluation metrics for each subject. It is important to highlight that the proposed 1D-CNN model demonstrated efficient performance across all datasets examined in this study. The average accuracy of the model is highest in the MAHNOB-HCI dataset, reaching 94.6%, and lowest in the DEAP dataset, with an accuracy of 84.2%. Additionally, the model successfully tackled the three-class problem in the SEED dataset, achieving an average accuracy of 91.7%.

Furthermore, the mean *p*-value (0.10) is highest in the MAHNOB-HCI dataset, indicating the significance of the selected feature vector compared to the original data. Although the average *p*-value (0.081) for the DEAP dataset is lower than that of MAHNOB-HCI, it is still satisfactory and demonstrates the importance of the selected features over the original ones.

In order to ensure a suitable training phase, we tested different numbers of augmented data and network designs. The experimental results determining the impact of augmented data are presented in Figure 6. As depicted in Figure 6, data augmentation proves to be more effective in the deep learning model. It considerably enhances the prediction accuracy of the 1D-CNN model. Specifically, doubling the data leads to improvements of up to 4.8%, 3.9%, and 3.5% for the DEAP, MAHNOB-HCI, and SEED datasets, respectively. This improvement can be attributed to the fact that deep neural network models require a larger amount of data compared to traditional machine learning models. In conclusion, data augmentation in EEG data is a complex task that requires careful consideration. In this experiment, a substantial amount of data was generated and added to the original dataset. However, augmented data did not uniformly improve results across all datasets. When increasing the original data amount threefold using our augmentation approach, we observed overfitting and degraded testing accuracy. This highlights the fact that that simply generating more data does not necessarily lead to better performance. There are optimal augmentation levels, beyond which additional synthetic data fails to provide new information and causes overfitting. The reliability of the classifier’s accuracy on data from different sources remains a significant concern in this task.

Table 8 presents the emotion recognition accuracy and Kappa coefficient using our proposed feature extraction method with and without data augmentation, compared to using all channels. Augmentation increased the maximum accuracy on MAHNOB-HCI to 97.61%, exceeding all-channel accuracy by 8.11%. For SEED, average accuracy was 95.27% with augmentation. DEAP accuracy improved by 4.81% to 89.02% with augmentation versus original data. The kappa scores were 0.841, 0.752, and 0.713 for MAHNOB-HCI, SEED, and DEAP, indicating substantial reductions in error versus chance levels. Overall, these results demonstrate that our proposed method of an emotion recognition and data augmentation approach considerably improves EEG-based emotion recognition accuracy and consistency.

Table 9 presents a concise overview and performance comparison between our proposed method and other existing emotion recognition methods. These methods are all deep learning models and have been considered for evaluation because they used the same dataset. Luo et al. [26] proposed three methods for augmenting EEG training data to improve the performance of emotion recognition models, utilizing deep generative models (VAE and GAN) and selective data augmentation strategies. These methods, namely cWGAN, sVAE, and sWGAN, were evaluated on SEED and DEAP datasets. Topic and Russo [40] presented a new model for emotion recognition using EEG signals, utilizing topographic and holographic representations of the signal characteristics. Deep learning was employed for feature extraction and fusion, resulting in improved emotion recognition performance on publicly available datasets. Zhang et al. [41] addressed the lack of interpretability in deep learning models for EEG-based tasks and explores the use of interpretable models to analyze CNN-based model SACNN in emotion recognition. By integrating brain science knowledge with interpretability analysis results, a new model was proposed, which demonstrated improved recognition accuracy on standard EEG datasets. Zhang et al. [42] presented a novel two-step spatial-temporal framework for emotion recognition based on EEG. The framework incorporated a hierarchical self-attention network to capture both local and global temporal information, reducing noise and localizing relevant segments. Additionally, the squeeze-and-excitation module and channel correlation loss were utilized to enhance spatial feature extraction and improve emotion recognition performance. Luo and Lu [43] addressed the challenge of limited EEG data for emotion recognition by proposing a Conditional Wasserstein GAN (CWGAN) framework. By leveraging generative adversarial networks, realistic EEG data in differential entropy form were generated and evaluated using quality indicators. Overall, the proposed method outperformed the methods from the literature and provided sufficient grounds for the idea that using a deep-learning-based lightweight 1D-CNN model with limited number of channels can produce successful results. Therefore, the major contributions of this study are listed below:

- It has been observed that the proposed deep learning method performs better than methods in the literature, even without data augmentation.
- Generally, large datasets are expected for deep learning studies. Therefore, the improved performance after augmentation further proves this point.
- In deep learning, manual feature selection is not typically conducted. In this study, deep learning was performed after feature selection. Manually selecting features may have caused some information loss. However, the deep learning model still achieved successful classification.
- Generally, all channels are used for emotion recognition. Despite the fact that this work utilized a limited number of channels, the deep learning model produced successful results.
- Processing raw data can increase deep learning algorithm performance. Due to the small sample lengths in this study, we could process the raw data, which increased overall performance.
- Although each dataset alone was small, the reliability of the proposed method was evaluated using different datasets and increasing the data size through augmentation on each dataset separately.

To summarize, Table 9 presents a comprehensive comparison between our proposed study and existing state-of-the-art methods in the literature, focusing on EEG-based techniques applied to the DEAP, MAHNOB-HCI, and SEED datasets. Our proposed approach outperforms other methods in terms of classification accuracy while also reducing computational overhead. By utilizing a 1D-CNN model and selecting high-quality features from specific channels, our method achieves simplicity and effectiveness. The results in Table 6 demonstrate that our model significantly improves precision compared to the previously employed model using the same datasets.

## 5. Conclusions

In this study, we tackled two key challenges in emotion identification from EEG signals: the high dimensionality problem and the scarcity of available data. To address these issues, we employed channel and feature selection methods to reduce the dimensionality of the EEG data and employed data augmentation using graph-EMD. The implemented approach exhibited superior accuracy on the proposed 1D-CNN model, highlighting the significance of addressing data scarcity in neural network models. The model was validated using DEAP, MAHNOB-HCI, and SEED datasets, demonstrating that our feature selection method effectively enhances overall classification performance while minimizing computational costs. Additionally, the proposed model offers notable improvements in feature extraction capabilities, while channel selection aids researchers in exploring the understanding of emotions through selective channels for precise emotion recognition. These results demonstrate the potential for a cost-effective IoT EEG device to enable accessible emotion recognition. By selectively extracting clean and relevant neural signals, our proposed methods can enhance the feasibility of real-world EEG acquisition. Our system provides an efficient and accurate pipeline from targeted EEG input to emotion state predictions. Moving forward, future research directions will explore further enhancements to the emotion analysis framework by integrating multiple neural networks. Considering the reduced testing and training time of the proposed model, it would be interesting to evaluate its performance across multiple emotion categories. Also, further analysis is needed to determine ideal augmentation factors for each dataset. While augmentation generally helped, more data did not linearly translate to accuracy gains. Additionally, this study was focused on small sample size for the sake of simplicity and reduced complexity; therefore, it is required to investigate the increased sample size for preprocessing to observe its impact on the performance.

## Figures and Tables

**Table 1:** Comparison of characteristics between all three datasets under discussion.

Characteristics	DEAP	MAHNOB-HCI	SEED
Participants	32	27	15
Trials	40	20	15
Channels	32	32	62
Sampling rate (Hz)	128	256	128
Affective states	Valence Arousal Dominance	Valence Arousal	Positive Negative Neutral
Rating scale range	1–9	1–9	-

**Table 2:** Size of the feature vector obtained at step of proposed method for each subject.

	Raw EEG	Channel Selection	Feature Selection
DEAP	32 × 7680	10 × 7680	10 × 5120
MAHNOB-HCI	32 × 15,360	10 × 15,360	10 × 10,240
SEED	62 × 30,720	10 × 30,720	10 × 5120

**Table 3:** Hyperparameters of the proposed 1D-CNN model.

Parameter Type	Range of Values	Selected Value
Number of 1DConvolution Layers	3–8	4
Number of filters	16–128	64, 32, 32, 16
Size of kernel	1 × 1–7 × 1	5 × 1, 5 × 1, 3 × 1, 1 × 1
Batch size	32–128	64
Learning rate	0.0005–0.01	0.005
Momentum	0.1–0.9	0.9
Dropout	0.25–0.5	0.25
Number of epochs	50–200	100

**Table 4:** Performance of the proposed method on DEAP dataset.

Subject	Accuracy	Precision	Specificity	Recall	*F*1-Score	*p*-Score
Subject 1	96.1	77	89	90	0.89	0.091
Subject 2	89.9	99	75	76	0.83	0.098
Subject 3	85.9	75	77	83	0.81	0.0697
Subject 4	78.3	76	73	70	0.87	0.084
Subject 5	77.3	82	75	66	0.85	0.067
Subject 6	83.1	81	71	66	0.85	0.0748
Subject 7	86.4	95	91	92	0.87	0.083
Subject 8	83.2	92	74	77	0.84	0.0852
Subject 9	83.8	89	78	76	0.86	0.09
Subject 10	82.9	81	87	81	0.80	0.08
Subject 11	76.5	67	89	75	0.86	0.0849
Subject 12	87.2	91	79	72	0.89	0.084
Subject 13	83.8	71	90	86	0.8	0.0951
Subject 14	87.6	76	93	69	0.94	0.075
Subject 15	87.2	91	92	99	0.8	0.0732
Subject 16	85	73	74	68	0.79	0.089
Subject 17	83.8	86	73	70	0.84	0.085
Subject 18	84.6	69	91	86	0.87	0.079
Subject 19	81.8	71	75	90	0.9	0.08
Subject 20	83.3	94	66	76	0.91	0.069
Subject 21	84.1	85	79	82	0.87	0.09
Subject 22	84.9	89	78	92	0.81	0.076
Subject 23	86.03	68	65	86	0.82	0.072
Subject 24	81.12	76	77	84	0.78	0.0921
Subject 25	82.5	71	70	96	0.82	0.0695
Subject 26	87.3	94	77	84	0.9	0.09
Subject 27	82.52	85	95	74	0.83	0.088
Subject 28	80.37	85	81	64	0.84	0.07
Subject 29	85.61	94	98	98	0.87	0.087
Subject 30	82.28	94	88	79	0.95	0.075
Subject 31	83.84	93	92	78	0.89	0.076
Subject 32	85.25	82	78	78	0.83	0.0785
Average	84.2	83	81	80	0.85	0.081

**Table 5:** Performance of the proposed method on MAHNOB-HCI dataset.

Subject	Accuracy	Precision	Specificity	Recall	*F*1-Score	*p*-Score
Subject 1	95.3	93	89	90	0.89	0.072
Subject 2	97.6	92	96	93	0.89	0.078
Subject 3	97.4	94	89	93	0.91	0.0839
Subject 4	91.2	89	92	90	0.99	0.073
Subject 5	92.7	90	85	87	0.99	0.072
Subject 6	96.5	88	87	86	0.97	0.0794
Subject 7	91.1	96	93	94	0.91	0.067
Subject 8	93.1	97	89	90	0.93	0.0802
Subject 9	95.8	95	92	92	0.97	0.08
Subject 10	94.5	93	94	91	0.89	0.0812
Subject 11	92.5	94	98	95	1.00	0.073
Subject 12	96.7	92	92	98	1.00	0.0779
Subject 13	95	89	90	96	0.97	0.0793
Subject 14	96	90	93	99	0.99	0.79
Subject 15	92	88	94	82	0.91	0.0684
Subject 16	93.5	90	89	85	0.90	0.076
Subject 17	96.6	91	92	92	0.99	0.08
Subject 18	95.1	92	86	91	0.99	0.0858
Subject 19	96.1	95	90	89	0.94	0.076
Subject 20	94.2	94	92	94	0.99	0.0831
Subject 21	93.9	93	92	96	0.89	0.0792
Subject 22	95.7	94	93	93	0.96	0.087
Subject 23	93.2	82	89	95	0.89	0.083
Subject 24	96.2	88	92	90	0.94	0.086
Subject 25	95	98	89	88	1.0	0.08
Subject 26	95.6	91	92	92	0.95	0.082
Subject 27	92.6	92	92	90	0.95	0.0717
Average	94.6	92	91	91	0.95	0.10

**Table 6:** Performance of the proposed method on SEED dataset.

Subject	Accuracy	Precision	Specificity	Recall	*F*1-Score	*p*-Score
Subject 1	93.8	87	92	91	0.97	0.0975
Subject 2	91.4	91	85	86	0.97	0.0845
Subject 3	92.8	94	89	83	0.91	0.09
Subject 4	91.1	97	84	87	0.98	0.092
Subject 5	90.2	89	82	90	0.95	0.0945
Subject 6	92.5	96	87	89	0.95	0.0935
Subject 7	89.4	89	96	94	0.91	0.0965
Subject 8	94.6	88	93	89	0.93	0.094
Subject 9	90.4	93	99	89	0.98	0.095
Subject 10	91.1	91	92	95	0.86	0.093
Subject 11	89.6	93	97	98	0.98	0.091
Subject 12	92.7	97	91	96	0.96	0.0885
Subject 13	90.5	78	80	96	0.93	0.087
Subject 14	93.5	90	92	98	0.97	0.095
Subject 15	92.1	81	85	82	0.91	0.0806
Average	91.7	90	89	91	0.94	0.092

**Table 7:** Computational time comparison of proposed method with and without channel/feature selection method.

	DEAP			MAHNOB-HCI			SEED		
	Channels	Feature Vector	Time (s)	Channels	Feature Vector	Time (s)	Channels	Feature Vector	Time (s)
CS+FS+1D-CNN	10	51,300	22	10	102,400	46	10	51,300	22
CS+1D-CNN	10	245,760	189	10	491,520	221	10	409,600	249
FS+1D-CNN	32	524,288	303	32	1,048,576	629	62	2,539,520	1066
1D-CNN	32	2,621,440	1100	32	5,242,880	1274	62	10,158,080	1675

**Table 8:** Comparison between all channels and selected channels in terms of accuracy with/without augmented data and Kappa coefficient for EEG classification.

	Original + Augmented	Original (10 Channels)	All Channels	Kappa
DEAP	89.02	84.21	81.06	0.713
MAHNOB-HCI	97.61	94.65	89.50	0.841
SEED	95.27	91.72	88.30	0.752

**Table 9:** Comparison between the results of the proposed method and the state of the art methods from literature according to various factors and the number of datasets used.

	Augmentation	DEAP			MAHNOB-HCI			SEED		
		Accuracy	Channels	Classes	Accuracy	Channels	Classes	Accuracy	Channels	Classes
Proposed	Yes	89	10	4	97.6/10	10	4	95.3	10	3
Luo et al. [26]	Yes	59.1	32	4	-	-	-	93.5	62	3
Topic and Russo [40]	No	77.72	32	4	-	-	-	88.45	62	3
Zhang et al. [41]	No	83	32	10	71	10	2	89	10	2
Zhang et al. [42]	No	77.03	32	4	81.37	32	4	79.56	62	3
Luo and Lu [43]	Yes	78.17	32	4	-	-	-	86.96	62	3

## Data Availability

Publicly available datasets were analyzed in this study. DEAP, MAHNOB and SEED can be found here respectively: https://www.eecs.qmul.ac.uk/mmv/datasets/deap/download.html (accessed on 10 July 2020), https://mahnob-db.eu/hci-tagging/ (accessed on 7 July 2020); https://bcmi.sjtu.edu.cn/home/seed/index.html (accessed on 13 July 2020).
